# The Evolution of Bladder Augmentation: From Creating a Reservoir to Reconstituting an Organ

**DOI:** 10.3389/fped.2014.00010

**Published:** 2014-02-10

**Authors:** Roman Jednak

**Affiliations:** ^1^Division of Pediatric Urology, The Montreal Children’s Hospital, McGill University Health Centre, Montreal, QC, Canada

**Keywords:** myelomeningocele, neurogenic bladder, bladder augmentation, tissue engineering

## Abstract

Bladder augmentation was first described in 1899. The goal at the time was to establish the ideal method to create a simple capacious reservoir for the safe storage of urine. That simple idea has over the last 100 years grown into one of the most dynamic areas in Pediatric Urology. Creative minds and hands from individuals in multiple disciplines have led us from creating a reservoir to the threshold of recreating a functional organ. In this review, we look at the historical evolution of bladder augmentation and how it exponentially grew in scope from those initial descriptions of intestinocystoplasty to the work being reported today in the field of tissue engineering.

## Introduction

The evolution of bladder augmentation is now well over 100 years old. The technique was first applied to patients and reported independently by both Rutkowski and Mikulicz in 1899. Each described ileocystoplasty, either as a single or staged procedure, in a patient with bladder exstrophy (1, 2). Rutkowski felt that with bowel we had out ideal material for cystoplasty while Mikulicz cautioned that it was the final functional outcome obtained by the procedure that would determine its value. Since these initial reports, we have been witness to a myriad of technical and scientific innovations in people’s attempts to develop the optimal technique and material for bladder augmentation. The creative minds and skillful hands of countless people have taken us far beyond those initial goals of simply trying to create a larger reservoir and have moved us to the field of tissue engineering and bladder regeneration. Oliver Wendell Holmes Jr. felt that “man’s mind stretched to a new idea, never goes back to its original dimensions.” Since those initial papers published by Rutkowski and Mikulicz more than a century ago, the field of bladder augmentation has certainly held true to this.

## Enterocystoplasty

### Ileo- and colocystoplasty

The objective of bladder augmentation is to create a low-pressure storage reservoir of sufficient capacity to preserve upper urinary tract function and maintain or establish urinary continence when maximal medical therapy is unsuccessful. In this respect, bladder augmentation using ileum or colon reconfigured to create a spherical final configuration has proven to be a reliable means of increasing bladder capacity and reducing bladder pressures (3–8). However, the incorporation of these intestinal segments into the urinary tract soon highlighted the fact that patients were at risk for a number of long-term complications attributable to the presence of intestinal mucosa (9). This was directly attributable to the differences in absorptive and secretory properties between urothelium and bowel mucosa. The typically impermeable characteristics of the urothelium were being altered. Furthermore with the young patient population usually being treated, the long-term exposure to these complications heightened the concern. The complications most commonly observed included mucus production, bacterial colonization, electrolyte imbalances, metabolic acidosis, somatic growth retardation, vitamin B_12_ deficiency, and the formation of bladder calculi (10–20). The potential to develop a malignancy became a significant concern and a number of reports were published to this effect over the years (21–23). A recent analysis by Higuchi et al. however evaluated 153 patients with an ileal/colonic cystoplasty and a matched control population. The authors found that patients with congenital bladder dysfunction undergoing ileal/colonic bladder augmentation do not appear to increase their risk of bladder malignancy over the inherent risk associated with the underlying congenital anomaly alone. It seems that the congenital dysfunctional bladder alone represents the increased risk for malignancy (24).

### Gastrocystoplasty

Sinaiko first described gastrocystoplasty using stomach for urinary diversion in a dog model (25). The technique was first introduced clinically by Leong and Ong (26, 27). In North America, gastrocystoplasty was introduced as an alternative to colon or ileum in children with chronic renal failure and azotemia as a direct result of its natural acid secreting ability. This did not worsen the metabolic acidosis as was typically seen with ileum or colon (28). The technique was also useful when bowel resection was not an option, as is the case with short-bowel syndrome. Mucus production was less problematic and the acidic urine could also reduce bacterial colonization and the incidence of urinary tract infections. Specific complications have become apparent however and include intermittent hematuria, metabolic alkalosis, and the hematuria–dysuria syndrome. This is characterized by bladder or urethral pain and hematuria in the absence of infection (29, 30). Furthermore, children with incontinence or renal insufficiency/oliguria tend to experience more problems with the hematuria–dysuria syndrome. Since the hematuria–dysuria syndrome does not always respond well to histamine antagonists, this may pose a significant problem in children with normal bladder and urethral sensation. An additional concern is that the inherent metabolic acidosis in patients with renal insufficiency reverses with renal transplantation. The presence of a gastrocystoplasty in this situation creates a tendency toward metabolic alkalosis; a problem significantly more challenging to manage medically. The frequency and severity of complications after gastrocystoplasty has resulted in the procedure losing favor and has led some to recommend abandoning it altogether (31). The majority of malignancies have been reported in patients undergoing enterocystoplasty but malignancies following gastrocystoplasty have been reported (32–36). One such report observed a 14- to 15-fold increased risk for the development of bladder cancer over standard norms (36). To date there have been no studies examining whether augmentation gastrocystoplasty increases the risk of bladder malignancy over that inherent to a congenitally abnormal bladder however.

## Urothelial Preserving Augmentation Techniques

Marucci and Shoemaker described the characteristics of the deal segment for bladder augmentation/substitution in 1955 (37, 38). Conceptually, they felt that the ideal material should:

- be easily available
- allow large areas to be potentially used
- be easily mobilized without jeopardizing its blood supply
- allow regrowth of urothelium
- have sufficient pliability to assume the shape of the bladder
- have an inherent functioning bladder layer
- not absorb electrolytes or nitrogenous wastes of the urine
- not produce mucus or irritating substances.

This spurred the reporting of numerous creative techniques for urinary reconstruction. Most of these procedures used part of the alimentary tract for this purpose. Several alternatives to the use of bowel or stomach were proposed. All of them had the common goal of eliminating or diminishing the problems associated with the incorporation of gastrointestinal mucosa into the urinary tract. The variety of natural materials used has included peritoneum (39), omentum (40, 41), fascial free grafts (42), lyophilized human dura (43), bovine pericardium (44) fresh placental membranes (45), and preserved bladder grafts (46), as well as synthetic materials such as polyvinyl sponge (47), gelatin sponge (48), and teflon-felt (49). These are now simply historical anecdotes and will not be discussed further but the extensive work generated does highlight the creativity that has gone into establishing a clinical alternative to enterocystoplasty.

It was clear from the wide experience with intestinal augmentation that gastrointestinal tissue has fallen short in achieving these ideals outlined by Marucci and Shoemaker on a number of levels. Certainly the ability to create a capacious low-pressure reservoir could reliably be achieved. The associated cost however was the exposure to a variety of serious complications secondary to the inherent physiologic differences between bowel mucosa and bladder epithelium. This has over the years given rise to a number of innovative and clinically applicable techniques aimed at preserving the urothelium in order to maintain the physiologic integrity of the reconstructed bladder epithelium.

### Ureterocystoplasty

The natural characteristics of the ureter, namely its elasticity, a wall of smooth muscle and a urothelial lining made it a very attractive augmentation material. The first clinical report of ureterocystoplasty was published in 1973 by Eckstein and Martin who described the use of a longitudinally incised ureter to augment the bladder of a 7-month-old infant (50). The technique was revived enthusiastically in the early 1990s by four independent groups and was attractive on several levels (51–54). Since the urothelium was preserved, acid–base disturbances and mucus production were not a problem. Furthermore, the procedure could be performed using an exclusively extraperitoneal approach. The inherent drawback however was the limitation in its applicability. Suitable patients had to have a dilated ureter serving a non-functioning kidney. Technique had to be meticulous in order to preserve the renal pelvis and ureteral blood supply. Observations derived from the outcomes of enterocystoplasty dictated reconfiguration of the segment should be in the form of a U-shape in order to create a sphere and maximize the final storage volume. The early results were favorable with Landau and colleagues showing urodynamic outcomes to be comparable to those of ileocystoplasty in an age-matched control group. Specifically, the mean pressure specific volume at 30 cm H_2_O was found to be 413 ml for the ileocystoplasty and 380 ml for the ureterocystoplasty groups respectively. Bladder compliance was found to be normal in 87.5% of patients following ureterocystoplasty (55). Similarly, favorable urodynamic parameters have been reported by Nahas et al. in a cohort of patients having undergone ureterocystoplasty and renal transplantation (56). At mean follow-up of 50 months, no graft loss was reported. They did not however, report the incidence of graft dilatation in their patients. Johal et al. examined 17 patients undergoing ureterocystoplasty at a mean follow-up period of 4.5 years (57). Urodynamic evaluation showed an improvement in mean bladder capacity from 125 to 292 ml and in mean compliance from 2.1 to 16.2 ml/cm H_2_O. Four patients required reaugmentation.

Other groups have shown less favorable results and have attempted to define criteria for successful ureterocystoplasty. Hussman et al. prompted by their overall disappointing experience with ureterocystoplasty and the lack of definitive criteria for patient selection, undertook a multi-institutional retrospective study examining the outcomes in 64 patients. Specifically, they recommended that patient without reflux and either a single or duplex collecting system could benefit from ureterocystoplasty if the ureter was at least 1.5 cm in diameter. Patients with reflux improved only if the compliance of the total system was normal or mildly non-compliant (compliance greater than 20 ml/cm H_2_O) prior to augmentation. Detubularization and augmentation of the bladder with a refluxing megaureter in the presence of poor compliance was found to be of little benefit (58). Podesta and colleagues similarly showed less favorable urodynamic outcomes with ureterocystoplasty in comparison to enterocystoplasty with both overall final capacity and compliance being better in the enterocystoplasty group (59).

The limitations of ureterocystoplasty in terms of applicability remain an inherent issue. Early experimental work naturally tried to address this. The earliest such report used a rabbit model and described progressive dilatation of a normal sized ureter over a 30-day period by means of saline injections through a subcutaneously implanted injection port. Ureteral units were dilated by at least 10-fold and augmentation cystoplasty performed with these dilated ureteral segments increased bladder capacity by an average of 260% (60). Later a similarly successful study evaluating ureteral tissue balloon expansion using a pig model supported these results (61). Although an interesting experimental work, the difficulty faced with attempting the clinical application of techniques such as these has yet to be circumvented. In an attempt to widen the clinical application of ureterocystoplasty to a broader group of patients, developments in surgical technique have been reported that use segments of ureteral tissue derived from functioning kidneys (62–67). Techniques such as transureteroureterostomy (62) or the creation of an ileal (65) ureter have been described to maintain drainage of the upper urinary tract. These may be technically challenging and expose the upper urinary tract to a new spectrum of potential complications such as obstruction, stones, metabolic alterations, and possibly malignancy.

Overall the outcomes of ureterocystoplasty have been favorable but the technique is not universally applicable and the ability to reliably augment the bladder to the degree achieved by enterocystoplasty without reconstruction of the upper urinary tract has not been conclusively demonstrated.

### Autoaugmentation cystoplasty

The novel concept that excision or incision of the diseased detrusor could be performed to create a urothelial diverticulum that could serve to augment the bladder was first described by Cartwright and Snow in 1989 (68, 69). Like ureterocystoplasty, the motivating factor behind its development was the creation of a capacious and compliant reservoir totally lined by urothelium. In the fact that the procedure was extraperitoneal, had a shorter operative time and did not affect the gastrointestinal tract added to its appeal. The benefits of preserving the urothelium were clear but with the increasing clinical experience that followed in subsequent years, the reported urodynamic outcomes have varied. Some authors have described only slight improvements in bladder capacity, whereas others have noted improvements in capacity with persistently high bladder pressures (70–72). In the series reported by Stothers et al. (71) simple vesicomyotomy was performed in their patients after earlier experimental work had suggested detrusor excision offers no advantage over detrusor incision (73). That same experimental work has shown that histologically the autoaugmented area was characterized by a fibrous infiltrate with an increase in thickness of 500% in comparison to the thickness immediately after augmentation. Similarly, Garibay et al. noted the development of a thin layer of fibrosis between the bladder mucosa and peritoneum, which possibly accounted for the observed lack of improvement in bladder capacity in their animal investigation (74). Cartwright and Snow reported on a combined series of 25 patients several years after their original publication and described their outcomes as encouraging but unpredictable. Success was judged as good in 52%, acceptable in 28%, and poor in 20%. Those patients requiring reaugmentation were found to have a layer of collagen laid down over intact urothelium in the area of the original procedure (75).

Several series have reported poor success rates in patients with neurogenic bladder dysfunction (76–79). MacNeily et al. found the short term outcomes promising but unfortunately not durable (79). Twelve of 17 patients were considered failures as a result of ongoing incontinence or ongoing upper tract deterioration. Marte et al. suggested that the mechanism of action with autoaugmentation might be a direct effect on the ability of the detrusor to contract rather than augmentation of bladder volume. The authors felt that the patients most likely to benefit are those with primary detrusor instability and a high leak-point pressure (78). Gurocak et al. reviewed the long-term results of autoaugmentation looking at 150 published studies (80). They found that better compliance after autoaugmentation procedures seems to be less pronounced and of shorter duration than that of conventional enterocystoplasty. The best candidates seemed to be those with a close to normal capacity and poor compliance. It appears that the best outcomes can be expected in those patients with good capacity (greater than 75% of that expected) but poor compliance.

Bladder autoaugmentation offers an extraperitoneal surgical approach, low morbidity, and urothelial preservation but has unfortunately not lived up to the initial enthusiasm it generated. Over the years its general applicability has narrowed in scope with the clinical experience suggesting that best results seem to be obtained in a select group of patients. Furthermore, the overall increases in bladder volume that can be reliably obtained do not approach those obtained by conventional enterocystoplasty and in the long-term the outcomes do not appear as durable.

### Seromuscular enterocystoplasty

The efforts to develop the ideal material for bladder augmentation made significant advances with elimination of the metabolic complications associated with the incorporation of bowel into the urinary tract. Both ureterocystoplasty and autoaugmentation made use of the ideal tissue to eliminate these issues and were significant technical developments. They eliminated most of the major disadvantages of enterocystoplasty. Unfortunately clinical experience fell short in terms of the general applicability of these techniques and the reliability of the degree of augmentation that could be achieved. Any new technique should continue to be reliable in addressing the primary clinical goal in question and that is the creation of a capacious low-pressure reservoir to protect the upper urinary tract and establish urinary continence.

Seromuscular enterocystoplasty was developed with the intent of combing the benefits of urothelial preservation with those of intestinal augmentation. Urothelial preservation would eliminate the metabolic complications associated with enterocystoplasty and the intestinal segment would more reliably augment the bladder. The early experimental work will not be reviewed here in depth but it would suffice to say that the results were encouraging but viewed with cautious optimism. The early experimental work had been performed using denuded segments of bowel or denuded segments grafted with a patch of bladder mucosa to augment the bladder after total or subtotal cystectomy (37, 38, 81, 82). After a critical review of the technique by Koontz et al. where they concluded that the complication rate was high and the clinical application has limited usefulness work on the technique was largely abandoned. He felt that the clinical application has limited usefulness (83). The idea was resurrected in 1988 when Oesch, using a rat model, demonstrated successful overgrowth of the denuded bowel surface with urothelium when demucosalized segments of cecum were anastomosed to the bladder following subtotal cystectomy (84). Pippi Salle et al. attempted to duplicate these experiments in dogs but the colonic patches from which the mucosa had been stripped underwent severe contraction and fibrosis in contact with urine (85). Similar outcomes using a rat and dog model were obtained by Gonzalez and his team (86, 87). Urothelial regrowth would occur in a small animal model but not in the large animal model. The poor experimental results obtained with partial detrusorectomy to increase bladder capacity prompted the development of a dog model in which the detrusor was removed with preservation of the urothelium (88). The resulting diverticulum was then covered with a reconfigured segment of sigmoid colon from which the mucosa had been removed. Preservation of the bowel submucosa was essential to avoid contraction of the colonic segment. Histological evaluation revealed that the composite organ was composed of the seromuscular and submucosal layer of the colon covered with transitional epithelium. Lima et al. described a similar operation and also obtained encouraging results (89). They subsequently went on to develop a dog model for seromuscular ileocystoplasty whereby a patch of demucosalized ileum was used to replace the whole bladder. Bladder configuration was maintained by the use of a silicone balloon placed within the augmented bladder and similarly encouraging results were obtained (90, 91). Dewan and his group took a different but similarly successful approach and used demucosalized stomach patches in combination with urothelial preservation (92).

Dewan and Stefanek published the first clinical application of seromuscular colocystoplasty. They reported on a 9-year-old girl with myelomeningocele who achieved an increase in bladder capacity from 40 to 213 ml, an increase in compliance from 1 to 14 ml/cm H_2_O, and was totally continent on a regimen of clean intermittent catheterization every 3 h (93). Overall, the clinical experience has been positive as reported by a number of other groups. The clinical experience with seromuscular colocystoplasty lined with urothelium (SCLU) in 16 patients reported by Gonzalez and his group showed a 2.4-fold increase in bladder capacity. Two patients failed augmentation and required ileocystoplasty. Two of thirteen patients with incontinence remained incontinent and there were no patients with metabolic acidosis or electrolyte abnormalities. Urothelial biopsies in 10 patients showed exclusively urothelium in 7 (94). Jednak et al. reported on another group of 32 patients followed for a mean period of 1.6 years and showed that total bladder capacity and capacity at 30 cm H_2_O increased by 1.8- and 2.4-fold respectively. This was irrespective of how bladder capacity was calculated. Achieving continence generally required a bladder neck procedure. All but two patients achieving continence had undergone a bladder outlet procedure prior to, at the time of, or following augmentation. An hourglass deformity developed in 22%, 12.5% failed augmentation, and 6% developed bladder calculi. New onset or increased hydronephrosis and reflux were seen in 10 and 15% of evaluated renal units respectively. The majority of patients developing upper tract dilatation either failed augmentation or developed an hourglass deformity. Colonic mucosal regrowth to varying degrees was seen in five of seven interpretable biopsies. Two patients had only transitional epithelium. There were no bladder perforations, metabolic abnormalities did not develop, and mucus production was not clinically significant (95). Bandi et al. reported longer-term outcomes on some of the patients undergoing SCLU in the early clinical experience of Gonzalez and his group. These were compared to a group of children undergoing either colocystoplasty or ileocystoplasty. They found the SCLU patients were at a decreased risk of bladder perforation and bowel obstruction but were otherwise not devoid of other long-term complications such as vesicoureteral reflux, bladder calculi, and augment failure. There was no long-term metabolic data provided (96).

The main prerequisite for success appears to be that bladder distension be maintained in the post-operative period. This appears to be the determinant of hourglass deformities as well as outright failures. As a consequence, the procedure is not recommended in those patients undergoing any additional procedure that requires opening the bladder thus limiting its applicability to patients requiring creation of a continent catheterizable channel at the same time. It has been recommended that continent catheterizable channels if required be done as a second procedure (97).

The clinical experience with both colon and ileum has been outlined with some variation in technique by Lima and his group in several reports. After initially using the urothelial preserving technique in combination with sigmoid colon (89), the group opted to abandon urothelial preservation and simply distend the augmented bladder post-operatively via placement of a silicone intravesical mold. The ureters were diverted for a 2-week period at which point both the ureteral stents and bladder mold were removed. The first report published looked at the results with colon in 24 patients. Overall the mean improvement in bladder capacity was fourfold. In absolute terms however the preoperative bladder capacity was 100 ml or less in all but two patients and post-operatively 15 of 24 patients achieved a bladder capacity of 200 ml or less. Compliance was reported as improved but specifics were provided. No mucus production was noted. Continence rates were excellent for patients with neurogenic bladder but disappointing for patients with bladder exstrophy. Data with respect to metabolic evaluation and upper tract deterioration were not reported (98). The only additional procedure performed at the time of augmentation in some of the patients was the placement of an artificial urinary sphincter.

Lima et al. later reported on their clinical experience in 25 patients undergoing ileal seromuscular cystoplasty using the same silicone mold technique (99). It is not clear if any of the patients underwent concomitant procedure at the time of augmentation. The overall percent increase in capacity was encouraging but the majority of patients had a reported final augmented capacity of less than 200 ml and changes in the upper urinary tract over time in this group of patients was not reported (99). Lima and his group also found that bladder distension is critical to achieving optimal results. Patients undergoing seromuscular augmentation without placement of an intravesical mold for the post-operative period typically did worse.

The clinical experience with gastric segments for seromuscular enterocystoplasty has been variable. The initial report on a very small group of patients by Dewan and Stefanek found that increases in bladder volume and compliance were not as impressive as with enterocystoplasty (100). The results were evaluated at 3 and 12 months post-operatively. Carr et al. reported variable results in a group of 13 patients with a mean follow-up of 50 months (101). They described their results as good in five, fair in four, and poor in four. Several techniques were used to ensure coaptation of the seromuscular segment and the urothelium in this group. These included simply the placement of a Jackson-Pratt drain between the seromuscular and urothelial layers, maintaining the bladder in a semi-distended state by adjusting the height of the Foley drainage bag, or the placement of an intravesical balloon to mechanically keep the bladder distended. It is not clear if the outcomes showed any relationship to the type of technique used to maintain coaptation in the post-operative period. As with other groups ancillary procedures were performed using an extravesical technique.

Seromuscular enterocystoplasty has certainly come closest to achieving augmentation with a tissue that maintains urothelial integrity and has a supporting muscular layer. At least one study however has suggested that there are no long-term differences in terms of vesicoureteral reflux, bladder calculi, or augmentation failure. Although it would inherently seem that metabolic derangements would not be an issue even in the long-term, metabolic assessments have nor been consistently reported. Limitations have also come to light. The parameters for a successful outcome limit some surgical techniques to those patients in which no concomitant intravesical procedures needs to be performed at the time of augmentation. Other techniques require a prolonged post-operative period of ureteral drainage and bladder distension using a silicone mold. Furthermore it has not been reported as to how an additional procedure such as ureteral reimplantation or the creation of a catheterizable channel are reliably performed using the silicone mold technique.

## Bladder Tissue Engineering

Urinary tract reconstruction has evolved to the point where it is now possible to effectively establish continence and prevent upper urinary tact deterioration. The goal of simply improving the bladder’s function as a reservoir has evolved beyond this as the limitations and complications associated with incorporation of gastrointestinal tissue into the urinary tract has become apparent and the physiologic mechanisms underlying these understood. Remarkable advancements in techniques for bladder augmentation have been developed initially with great enthusiasm but as clinical experience has accumulated all have been shown to have limitations to some degree. These have included a lack of general applicability, the use of tissues with limitations in availability, technical surgical challenges, and unreliable outcomes. Given the technological limitations in basic science at the time, the hope was at best to duplicate bladder tissue and urothelial physiology and not reconstitute the bladder. Through developments in tissue engineering, however, previously unattainable goals have become a possibility. For the most part, the early clinical advancements focused on limiting physiologic derangements associated with augmentation by attempting to maintain urothelial integrity since this was after all the source of the majority of complications. The potential to reliably restore the functional aspects of the detrusor were out of reach. They could be mimicked to a small degree but this fell far short of the normal state. We could not reproduce the characteristics of the intricate arrangement of smooth muscle, nerves, and blood vessels. The bladder is a complex organ composed of a specialized urothelium and a compliant detrusor. It functions as a low-pressure, high-capacity reservoir that can empty completely. This in turn is modified by and responds to direct conscious input. At the bladder level, this requires complex interplay that occurs on multiple levels including neural pathways, the bladder smooth muscle, the urothelium, and the sphincteric components of the bladder outlet. Developments in tissue engineering have allowed us to look toward the possible replacement of complex bladder function in situations where this ability has been lost. This has come about through progress at a multidisciplinary level with advancements in fields such as biology, medicine, and engineering (102, 103).

Tissue engineering has four requirements and those are the presence of adequate stem/progenitor cells, an appropriate extracellular matrix or carrier construct, an appropriate blood supply, and the presence of regulatory signals (104).

Two approaches can be used. The acellular approach makes use of scaffolds to enhance the body’s natural ability for self-repair. These assist in determining the direction of new tissue growth. This can pose a problem when the tissue from which regeneration is required is itself diseased. The cellular approach makes use of donor cells to seed the scaffold. This creates a variety of options from which cells can be derived. These can be autologous, allogeneic, or heterologous. Immunogenicity is a concern however. The best option remains autologous cells since this eliminates the possibility of rejection. Current research is focused on two areas: determining the most appropriate cells for bladder regeneration and designing the best scaffolds to reproduce the physiology and function of the tissue being replaced (105). The cells in particular should be readily available, easily harvested, and have the ability to regenerate a normal bladder. There are concerns that cells derived from a diseased bladder may not be able to revert to normal and as such the use of stem cells for this purpose is being extensively investigated (106, 107).

Scaffolds are tissues designed to support cell growth and can be natural or synthetic. Naturally derived scaffolds are derived form autologous, allogeneic, or xenogeneic tissues that are processed by chemical or mechanical means in order to remove the cellular components. Although stripped of their cellular element, they still maintain important extracellular matrix components such as collagen, fibronectin, laminin, glycosaminoglycans, and growth factors that can stimulate the regeneration of smooth muscle and urothelial cells by promote cell migration, growth, and differentiation. These matrices can still retain some of their cellularity however and can potentially expose recipients to immunogenic components (102). Synthetic scaffolds have been developed as an alternative. These are biodegradable polymers constructed into a mesh able to support and direct tissue in growth and proliferation. Their synthetic nature allows for the precise reproducibility of their composition. They do however lack the inherent biochemical components that direct tissue regeneration and neovascularization. The incorporation of these compounds into the scaffold is possible but regulating their release in a controlled fashion is an unresolved issue. The most commonly used compounds used for synthetic scaffolds are poly-α-esters such as poly(l-lactide), polyglycolic acid, and poly(lactide-co-glycolic acid) (104, 108).

The two natural products that have generated the most enthusiasm are porcine small intestinal submucosa (SIS) and bladder acellular matrix (BAM) (108). The results with each have been mixed however. Kropp et al. analyzed outcomes using a dog model in which they performed partial cystectomy and immediate grafting with acellular SIS. Evaluations times were set at 1, 3, 6, and 15 months. Histologically, all the tissue layers (mucosa, serosa, muscle) showed evidence of regeneration (109). Further assessment of the regenerated bladder tissue showed that the compliance was no different from that of control bladders and contractility studies showed a contractile response and innervation similar to that of a normal canine bladder (110). These were studies in normal bladders however. When similar studies were performed in damaged bladders following the removal of more than 90% of the detrusor, the results were less encouraging. There was limited bladder regeneration and the grafts were characterized by a central abundance of inflammatory cells and fibroblasts with few smooth muscle cells, poor vascularization, adhesions, and shrinkage. Some grafts even had bone and calcification at the graft site. It appeared that bladder regeneration in this model had limitations dependant on the state of the diseased bladder. Regeneration was dependant on the revascularization rate and the degree of pre-existing bladder injury (111).

Bladder acellular matrix was first used for bladder augmentation as an autologous acellular graft in rats by Probst et al. (112). The scaffold, prepared from the bladder dome of rats and consisting of primarily collagen and elastin, was used to immediately augment normal rat bladders following hemicystectomy. The animals were sacrificed at various time points with the latest being 20 weeks following surgery. The authors observed ingrowth of all bladder wall components as well as neural regeneration without signs of rejection. At 12 weeks post-operatively it was in fact difficult to distinguish the junction between native and regenerated bladder wall. Bladder capacity was preserved in the animals. Further evaluation went on to demonstrate that this regenerated tissue exhibited contractile activity in response to electrical field stimulation and a quantitatively identical pattern of response to nitric oxide and muscarinic, purinergic, and α- and β-adrenergic drugs. Histological and immunohistochemical studies confirmed the presence of receptors to these compounds (113). The subsequent evaluation of acellular BAM xenotransplants prepared form dog, rabbit, and hamster and grafted into rats had similarly encouraging results with the grafts promoting both functional and morphological regeneration of the bladder (114). The allograft technique was then transferred to a larger porcine model with promising initial results. At 8–12 weeks the grafts showed repopulation with all cellular components and on average had contracted by 25% (115). Outcomes at a longer time frame of 22 weeks post-operatively were disappointing however. Grossly there was macroscopic contracture of the grafts with only peripheral cellular repopulation. The central portions of the graft showed no evidence of smooth muscle bundles or urothelium (116). Once again the success seemed to be dependant on model size and to a significant degree on the rate and efficiency of neovascularization.

Seeding scaffolds with the cellular components necessary for bladder regeneration was developed as a means to promote a more rapid and diffuse distribution of cells across the scaffold which is then implanted into the host. Since the technique does not depend on tissue revascularization and ingrowth from the host, a more complete and efficient regenerative process might be expected in grafts of larger size. Work using both synthetic and natural grafts has been mixed. Early work published by Yoo et al. used seeded and unseeded allogeneic bladder submucosa and showed better outcomes in the seeded group which demonstrated normal cellular organization and less graft shrinkage (117). A different outcome was reported by Zhang et al. who found that seeded SIS grafts demonstrated limited regeneration and were characterized by shrinkage and adhesions (110). This was no different from the outcomes achieved using unseeded SIS grafts. Jayo and colleagues subsequently used synthetic scaffolds seeded with autologous urothelial and smooth muscle cells in dogs and observed better outcomes in the seeded versus unseeded groups. Characterization of the healing responses indicated that seeded grafts elicited an early healing process that led to regeneration of both mucosa and muscle whereas unseeded grafts stimulated a reparative response leading to incomplete tissue layer development (118).

The most advanced work has been generated by Atala and his group who were the first to reconstitute the bladder in a canine model undergoing trigone-sparing cystectomy (119). Transplantable neo-organs were cultured *in vitro* from synthetic scaffolds seeded with urothelial and muscle cells. Functional evaluations up to 11 months revealed a normal architecture, normal capacity to retain urine, and normal elastic properties. This transfer of this technology eventually resulted in a clinical human study in nine patients with myelomeningocele (120). The tissue used to seed the scaffolds was derived from the patients themselves. Patients received either a scaffold of collagen or a composite scaffold of collagen and polyglycolic acid. Three patients had an omental wrap to promote vascularization. Results were reported in seven patients with a mean follow-up of 46 months. Four patients were evaluated as far out as 49–61 months post-operatively. No metabolic derangements developed, renal function was preserved, there was no mucus production, and no calculi developed. All patients were incontinent preoperatively but it is not clear from the results how many patients achieved a socially acceptable level of continence post-operatively. No upper urinary tract follow-up post-operatively was reported. The improvements in bladder capacity were not analogous to those that can be achieved by standard enterocystoplasty techniques however. One patient developed a reduction in capacity and one improved from 139 to 480 ml. The improvement in capacity in the remaining patients ranged from 12 to 108 ml. Three patients had undergone an omental wrap to promote vascularization and these tended to have a better urodynamic outcome. The best improvements in compliance (2.8-fold) were seen in composite bladders wrapped with omentum. Pressure specific volumes at 30 and 40 cm H_2_O were not reported. One patient underwent a reoperative cystoplasty 4 years after surgery. This work was an important step in evaluating the transfer of tissue engineering technology to the clinical setting and the results can be viewed with some degree of optimism but certainly do not improve on the outcomes seen with many of the earlier developed augmentation techniques.

## Conclusion

The area of bladder augmentation has made remarkable advances over the last 100 years. Initially a purely surgical problem, finding the ideal tissue is now being addressed using a multidisciplinary approach. We have gone from harvesting tissue to tissue regeneration. We have progressed from simply improving a reservoir to sitting on the threshold of reconstituting a normal organ in humans. Despite our advances however the ideal tissue and technique for augmentation remains elusive. At present clinical experience suggests that standard bladder augmentation techniques offer the most reliable patient outcomes and should be the first choice for patients. The challenge to improve remains and the future holds promise for revolutionary new developments as research and technical innovations open the door to new frontiers.

## Conflict of Interest Statement

The author declares that the research was conducted in the absence of any commercial or financial relationships that could be construed as a potential conflict of interest.
